# Fatal hepatic failure following atorvastatin treatment: A case report

**DOI:** 10.1097/MD.0000000000033743

**Published:** 2023-05-12

**Authors:** Huajun Wang, Shiyi Liu, Chenjie Zhou, Ye Fu

**Affiliations:** a Department of Intensive Care Unit, The Affiliated People’s Hospital of Ningbo University, Ningbo City, Zhejiang Province, P. R. China.

**Keywords:** atorvastatin, case report, drug-induced liver injury, liver failure, statin

## Abstract

Atorvastatin is a commonly used statin for the treatment of hypercholesterolemia in people at high risk for coronary, cerebrovascular, and peripheral artery disease. However, fatal liver failure due to atorvastatin treatment has been rarely reported, especially during the very short incubation period.

**Patient concerns:**

A 63-year-old male patient was admitted due to unexplained chest pain. After admission, his liver function had decreased < 24 hours after taking 20 mg tablets of atorvastatin due to lacunar infarction, which was improved after drug withdrawal. The treatment regimen was restarted 15 days later, but within 16 hours, the patient developed multiple organ failure, including liver failure and renal failure.

**Diagnoses:**

The pathological results after 7 days indicated focal inflammatory necrosis, virus and autoimmune correlation tests were negative, which did not rule out drug-induced liver injury. Interventions: receiving artificial liver therapy, continuous renal replacement therapy and other organ support treatment.

**Outcomes:**

The patient died 18 days after admission.

**Lessons:**

Statin idiosyncratic liver injury is very rare, but the consequences can be serious.

## 1. Introduction

A drug-induced liver injury (DILI) is an important but rare adverse event that can lead tomild liver enzyme elevations, liver failure, the need for transplantation or even death.^[1]^ A large proportion of commonly used medications, in addition to herbal and dietary supplements, can cause liver injury. The most important drug groups are antibiotics, antituberculous medications, nonsteroidal anti-inflammatory drugs and immunosuppressants. Because many elderly and frail patients have basic cardiovascular and cerebrovascular diseases, severe infection is more common and more often complicated by other conditions requiring medication treatment. Once a drug-induced liver injury occurs, it is sometimes difficult to determine which drug is responsible for the injury. Statins are commonly used to treat hyperlipidemia, coronary artery disease and other atherosclerotic diseases because of their ability to lower cholesterol. Side effects limit the wide application of these drugs to a certain extent. Common side effects include neurological and neurocognitive effects, hepatotoxicity, renal toxicity, gastrointestinal, urogenital, reproductive, etc. A DILI is a relatively important side effect, with an incidence of approximately3%.^[2]^ A statin-induced liver injury is generally dose-and time-dependent and can be relieved after drug withdrawal, thus the probability of chronic hepatitis is very low. Although an idiosyncratic liver injury has a 75% risk of death or need for liver transplantation, itis not generally dose-dependent.^[3]^ Therefore, clinicians should be aware of and suspect fatal liver failure induced by atorvastatin.

## 2. Case presentation

**Figure 1.**
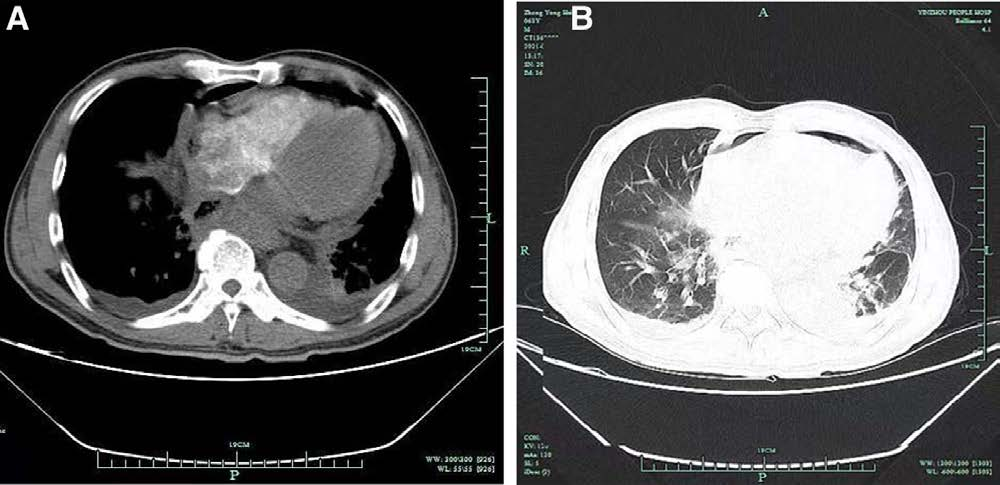
(A) Pericardial effusion with a little air, bilateral pleural effusion with left atelectasis, (B) Left lung ataxia, exudative changes in both lungs.

**Figure 2.**
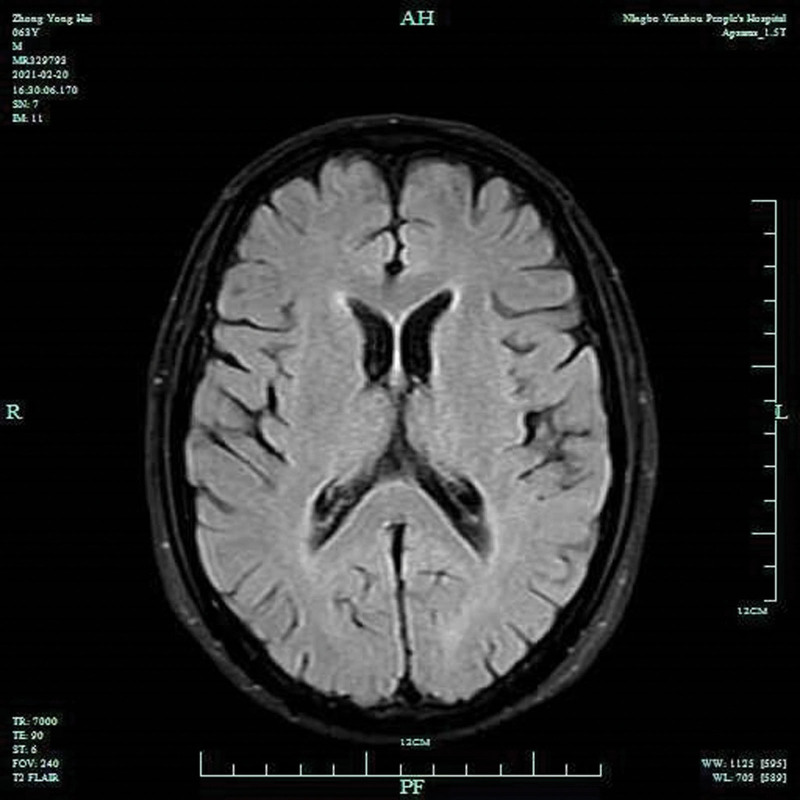
Spotty signal lesions were scattered in the white matter areas around the lateral ventricles on both sides.

A 63-year-old male farmer was admitted for “chest pain for 8 hours”. More than 2 years ago, the patient underwent thoracolaparoscopic radical resection of an esophageal carcinoma under general anesthesia, was treated with 2 courses of gimeracil and oteracil potassium capsules and followed up regularly. Eight hours before admission, the patient reported he experienced chest pain without obvious inducement, which was aggravated with respiratory movement and relieved after rest. No chest distress, shortness of breath, nausea, cough, expectoration, abdominal pain, or abdominal distension was reported nor observed at admission. Physical examination showed the following: body temperature, 38.0°C; pulse, 101 beats/minutes; respiration rate, 18 breaths/minutes; blood pressure,123/67 mm Hg; NRS score for left shoulder and back pain, 2 points; stable breathing; no icteric skin or sclera or bilateral supraclavicular lymph node enlargement; old surgical scars on the neck and chest, clear breath sounds in both lungs, with no obvious rales; flat and soft abdomen, no tenderness; and the liver and spleen were not palpable under the ribs. Chest CT showed the following: Postoperative changes in the esophagus; Arcuate gas shadow in front of the heart (suspected pneumomediastinum); A small amount of pericardial effusion, and; A patchy shadow in the anterior segment of the right upper lobe (Fig. 1). Routine blood tests showed a white blood cell count of 16 × 109/L and a neutrophil percentage of 92.6%. No abnormal findings were found on electrocardiogram Orin regard to myocardial enzyme levels. The patient was admitted due to the outpatient department for chest pain of an unknown origin. His differential diagnosis on admission included chest pain (further examination was required to rule out esophageal mediastinal fistula), pulmonary infection orpost operative complications of esophageal cancer. Following relevant examination to determine the diagnosis and treatment plan, the patient received piperacillin sodium and tazobactam sodium (4.5 g q8hIVGTT) to prevent infection, reduce phlegm, improve oxygen inhalation, and relieve pain relief and instructed to take appropriate precautions to prevent deep venous thrombosis. On the second day after admission, the cranial MR plain scan showed an acute lacunar infarction beside the anterior horn of the right ventricle and mild ischemic changes in the white matter (Fig. 2). On the third day of increased pericardial effusion, second-generation gene sequencing of pericardiocentesis fluid was performed and showed an infection with Rothiamucilaginosa, Veillonella sulfamethoxazole and Candida albicans. The antibiotics were adjusted to cefoperazone sulbactam 2 g q8 hours + tigecycline injection 50 mg Q12 hours + fluconazole injection 400 mg QD intravenous drip. On the 4th day, after neurology consultation, antiplatelet therapy with clopidogrel tablets and lipid-lowering therapy with atorvastatin tablets 20 mg QN PO was added.

**Figure 3.**
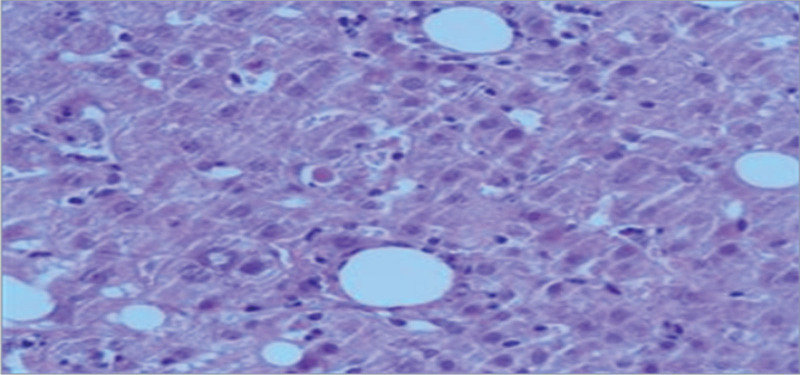
Focal necrosis and occasional apoptotic bodies were observed in hepatocytes, and pigmented granules were observed in some hepatocytes. There was a large amount of mixed inflammatory cells infiltrated mainly by mononuclear cells in the hepatic sinuses, a small amount of Kupffer cells phagocytic pigment granules, occasionally eosinophils, and focal perisinusitis was significant. The portal area was slightly enlarged, and there was more infiltration of mixed inflammatory cells, and a small number of phagocytic macrophages could be seen. Focal mild interfacial inflammation. Immunohistochemistry: HBsAg (−),HBcAg (−),α-SMA (−),CK7/CK19 showed positive bile duct epithelium, Fe staining (−) Cu staining (−).

Five days after admission, laboratory examination showed abnormal liver enzymes (Table 1), and no abnormalities were found on virological tests, autoimmune liver disease panels or abdominal imaging. Although the possibility of infection was considered, the possibility of DILI was not excluded, so meropenem 0.5q6 hour IVGTT was added to strengthen the anti-infection regimen. Drugs that may cause liver injury, such as tigecycline and atorvastatin, were stopped, and hepatoprotective drugs were added for treatment. To further identify the etiology of liver injury, the patient underwent liver biopsy and pathological examination on the 6th day. The pathological results after 7 days indicated focal inflammatory necrosis, virus and autoimmune correlation tests were negative, which did not rule out DILI (Fig. 3). After 8 days of treatment, the patient’s liver enzymes gradually returned to normal, and the infection was controlled. Atorvastatin calcium tablets was continued on the 15th night. Sixteen hours later, the patient developed significant nausea, vomiting with gastric contents and shortness of breath; blood gas analysis showed a partial pressure of carbon dioxide of 16 mm Hg and a partial pressure of oxygen of 95 mm Hg. The brain natriuretic peptide level was 1052 pg/mL and the D-dimer level was 3152 ng/mL, thus suggesting acute left heart failure, so cardiotonic and diuretic therapy was given. Blood gas analysis showed a partial pressure of carbon dioxide of 16 mm Hg, a progressive increase in blood lactate levels, unstable circulation under vasoactive vasopressors, and oliguria, so the patient was treated with continuous renal replacement therapy. The patient’s condition continued to deteriorate and laboratory tests showed signs of liver failure, soartificial liver therapy was performed at the bedside. The patient died 2 days after being transferred to a higher-level hospital (due to ineffective treatment).

**Table 1:** Changes of liver enzymes in patients after admission.

Days Liver enzymes	Day 1	Day 2	Day 5	Day 6	Day 7	Day 8	Day 9	Day 10	Day 11	Day 12	Day 13	Day 16	Day 17
ALT (u/L)	18	19	776	716	674	418	278	206	142	67	35	1286	4555
AST (u/L)	21	30	1112	518	316	105	62	61	33	33	28	1950	11,400
TBIL (μmol/L)	9.7	10.1	14.7	14	12.2	12.9	11.3	12.2	12.5	11.8	9.7	48.8	58
ITBIL (μmol/L)	7	7.2	8.6	10.5	8.5	5.4	4.6	5.1	5	5.8	5.3	28.6	37.9

## 3. Discussion

As the proportion of aging adults within the general population increases, the incidence of cardiovascular and cerebrovascular diseases is increasing yearly. Statins have been confirmed to significantly reduce serum cholesterol and triglyceride levels, reduce lipids in atheromatous plaques, reduce inflammation and improve endothelial function to effectively reduce the incidence and mortality of cardiovascular and cerebrovascular diseases, despite unknown side effects. They are considered to be relatively safe drugs and have become one of the most prescribed drugs in developed countries.^[4–6]^ The US Food and Drug Administration issued a statement on December 28, 2014, fully affirming the safety of statins and concluding that routine liver function tests are not required for administering statins because a statin-induced liver injury is rare and unpredictable,^[7]^ and recent studies have suggested that patients with cirrhosis are new candidates for statins.^[8,9]^ However, there are still case reports describing patients with fatal liver failure or needing liver transplantation as a result of taking statins, and although some patients recover after rescue treatment, hepatotoxicity is still an important concern for medical staff and patients.^[10]^ Statins are mainly discontinued because of side effects, which often occur inpatients of a certain sex, ethnicity, or with underlying diseases, such as hypertension and diabetes.^[11]^ Although the most common side effects are gastrointestinal reactions, such as constipation, abdominal pain, abdominal distension, and dyspepsia, rash, headache, rhabdomyolysis, and liver injury may also occur. However, it is generally believed that liver damage is limited and dose-dependent and can be relieved after discontinuation of the offending medication. The incidence of liver damage and the type of liver damage vary with different drugs; for example, cholestatic hepatitis is more common with atorvastatin, while hepatocellular hepatitis is more common with simvastatin.^[12]^ The patient suffered from severe infection and lacunar infarction. Liver function was impaired after the patient received an intravenous injection of tigecycline and oral atorvastatin calcium tablets but was improved after medication adjustment and infection control. Despite a significant temporal relationship, laboratory and imaging studies ruled out viruses, autoimmune liver disease, and obstructive biliary disease. The subsequent pathological finding suggest a DILI. The DILIN prospective study was conducted from 2004 to 2013 and found that the most common anti-infective agents were amoxicillin-clavulunate, isoniazid, nitrofurantoin, trimethoprim-sulfamethoxazole and minocycline.^[13]^ Is it reasonable to consider that this patient’s liver damage is related to tigecycline? Xiaoping reported a case of a drug-induced liver injury caused by intravenous tigecycline and found that bilirubin levels increased from 6.0 to 55% after tigecycline was administered, and the risk of drug-induced liver injury was mentioned in the manufacturer’s instructions.^[14]^ However, few reports have met the criteria for drug-induced liver injury, especially no reports of liver failure. The patient’s liver function was immediately impaired following the first dose of atorvastatin, and although his condition improved upon discontinuation, he still developed fatal liver failure 16 hours after the second dose. Combined with the known hepatotoxicity of statins and according to the Roussel Uclaf Causality Assessment Method (Tables 2) and Naranjo Causality Scale (Tables 3), Consider fatal liver injury with atorvastatin calcium tablets rather than tigecycline injection. Atorvastatin calcium tablet-induced liver damage is generally related to a prolonged course of treatment ora large cumulative dose within a period varying from 34 days to 10 years,^[10]^ however severe liver failure occurs in such a short time (in this case, it occurred within 16 hours), which has been rarely reported, with this being the second report to my knowledge. Early Li et al^[15]^ reported a case of mild-to-moderately impaired liver function 12 hours after the application of fluvastatin drugs, and the prognosis was good after drug withdrawal. The mechanism of statin-induced liver injury is unknown, and the possible cause may be autoimmune hepatitis induced by this class of drugs,^[16]^ but we examined autoimmune-related parameters and found no abnormalities. Thus, hepatic impairment due to this cause was tentatively excluded. It has also been suggested that a statin liver injury is associated with oxidative stress.^[17]^ However, because we lacked previous knowledge of this aspect, no relevant examinations were performed. A liver injury occurs very early in the course of treatment, is apparently not dose-dependent and is individual specific, similar to amoxicillin and clavulanate potassium.^[18]^ Therefore, a clinically significant statin-induced liver injury may have idiosyncratic and immune allergic mechanisms. The specific mechanism of statin-induced liver failure in this patient is still unclear. It is likely that individual susceptibility to statins, genetics and polymorphisms play an important role in the hepatotoxicity of statins.^[16]^A liver injury occurs very early in the course of treatment, is apparently not dose-dependent and is individual specific. Therefore, although statins are considered safe medications, it is still necessary to be aware of a potentially fatal liver injury based on this case and previous literature reports, especially for patients with acute liver disease, decompensated liver disease, or a history of suspected drug liver damage, for whom the use of these drugs should be avoided as much as possible. There is currently a lack of evidence supporting the reinitiation of a statin regimen or a reduced dosage regimen, and some data even suggest that it is relatively safer to replace statins with other drugs than to continue a statin regimen,^[10]^ but this hypothesis requires validation through more observational studies. However, due to insufficient attention in the early stage of treatment, a statin-induced liver injury was once mistaken for an infection-related liver injury, so the severity of a heterogeneous liver injury caused by atorvastatin was not fully recognized. In this patient, the later stage of liver failure progressed rapidly, with abnormal coagulation function and unstable vital signs, and liver transplantation was unfortunately not performed.

**Table 2:** Method for causality assessment of adverse drug reactions (RUCAM) Criteria.

Time to onset of the reaction		Score	
	Highly suggestive	+3	
	Suggestive	+2	2
	Compatible	+1	
	Inconclusive	0	
If incompatible, then case “unretated”			
If information not available, then case “insufficiently documented”			
Course of the reaction			
	Highly suggestive	+3	
	Suggestive	+2	2
	Compatible	+1	
	Against the role of the drug	−2	
	Inconclusive or not available	0	
Risk factor (s)for drug reaction			
	Presence	+1to + 2	1
	Absence	0	
Concomitant drug (s)			
	Time to onset incompatible	0	0
	Time to onset compatible but unknown reaction	+1	
	Time to onset compatible and known reaction	−2	
	Role proved in this case	−3	
	None or information not available	0	
Non-drug-related causes			
	Ruled out	+2	2
	Possible or not investigated	+1 to −2	
	Probable	−3	
Previous information on the drug			
	Reaction unknown	0	
	Reaction published but unlabelled	+1	
	Reaction labelled in the product’s characteristics	+2	2
Response to read ministration			
	Post live	+3	3
	Compatible	+1	
	Negative	−2	
	Not available or not Interpretable	0	
Or plasma concentration of the drug known as toxic + 3			
Or validated laboratory test with high specificity, sensitivity			
And predictive values			
	Positive	+3	
	Negative	−3	
	Not interpretable or not available	0	
	Total score	12	

**Table 3:** Naranjo causality scale for adverse drug reactions.

	Question/scoring yes/no/do not know or unavailable	Atorvastatin			Tigecycline			Fluconazole		
		Yes	No	NA	Yes	No	NA	Yes	No	NA
1	Are there previous conclusive reports on this reaction? 1/0/0	+1			+1			+1		
2	Did the adverse event appear after the suspected drug was given? 2/−1/0	+2			+2			+2		
3	Did the adverse reaction improve when the drug was discontinued or a specific antagonist was given? 1/0/0	+1			+1					0
4	Did the adverse reaction reappear when the drug was readministered? 2/−1/0	+2					0			0
5	Are there alternative causes that could have caused the reaction? −1/2/0			0			0			0
6	Did the reaction reappear when a placebo was given? −1/1/0		+2			+2			+2	
7	Was the drug detected in any body fluid in toxic concentrations? 1/0/0			0			0			0
8	Was the reaction more severe when the dose was increased, or less severe when the dose was decreased? 1/0/0			0			0			0
9	Did the patient have a similar reaction to the same or similar drugs in any previous exposure? 1/0/0	0			0					0
10	Was the adverse event confirmed by any objective evidence?	+1				0			0	
	Total	9			6			5		
	Scoring	Probable ADR			Possible ADR			Possible ADR		

Scoring > 9 – highly probable ADR; 5–8 – probable ADR; 1–4 – possible ADR; 0 – unlikely ADR.

## 4. Conclusions

In conclusion, statin idiosyncratic liver injury is very rare, but the consequences can be serious.

## Acknowledgments

We thank the staff and patients for their contributions and participation in the study.

## Author contributions

**Formal analysis:** Ye Fu.

**Investigation:** Shiyi Liu, Chenjie Zhou.

**Methodology:** Shiyi Liu.

**Writing – review & editing:** Huajun Wang.
